# Supplementary figures and images for: Finding driver mutations in cancer: Elucidating the role of background mutational processes
Source: PLoS Comput Biol. 2019 Apr 29;15(4):e1006981. doi: 10.1371/journal.pcbi.1006981 (PMC6508748; doi:10.1371/journal.pcbi.1006981)

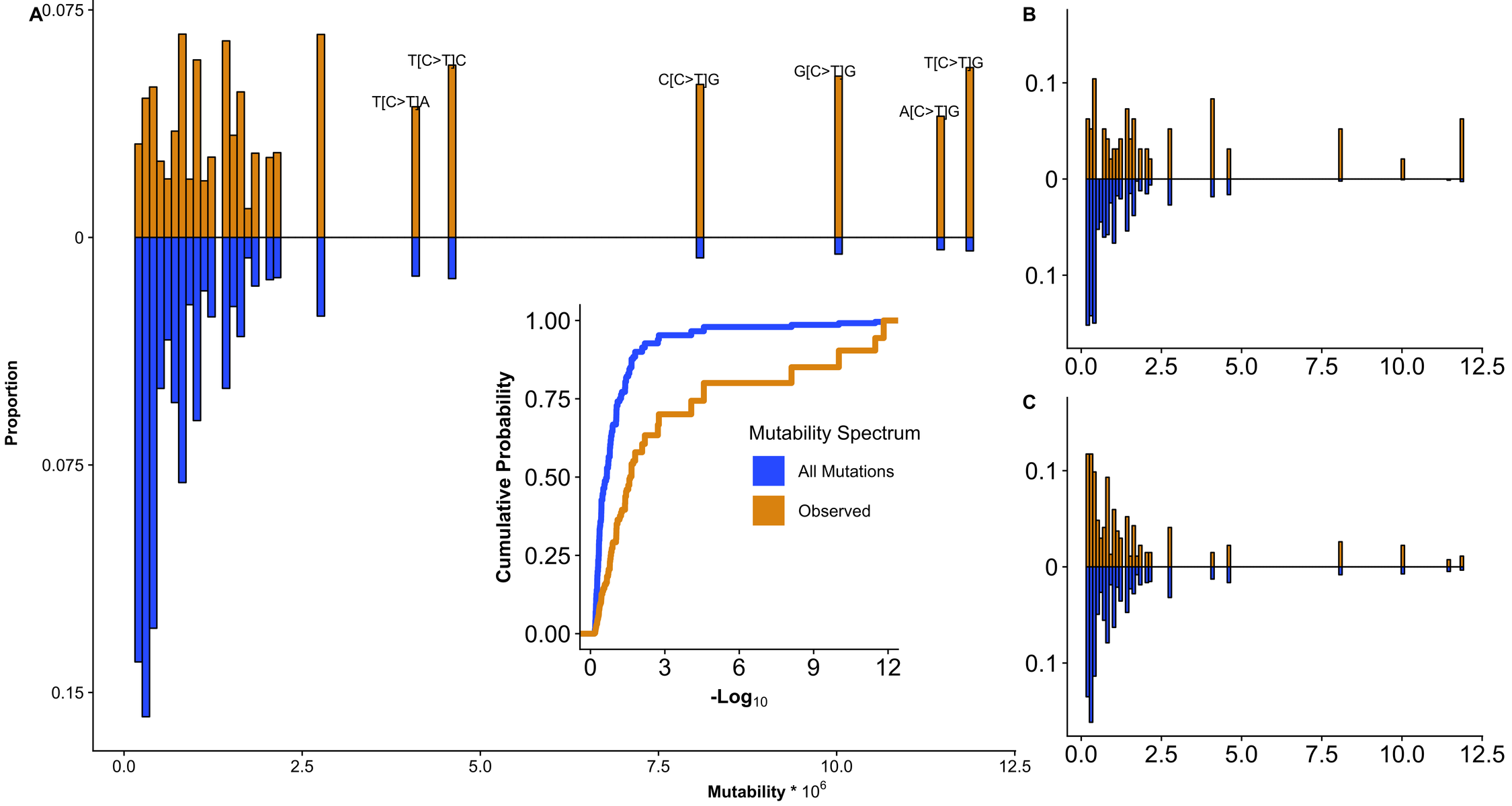

Supplement: S1 Fig — (A) Mutations from 520 cancer census genes; (B) CASP8 and (C) TP53 genes. Y-axis has been mirrored and shows the proportion of nucleotide mutations with the mutability given on the X-axis. For example, 5.6% of the 57,074 observed nucleotide mutations occurred at a site with the maximum pan-cancer nucleotide mutability of 1.18 x 10^−5^, despite the fact that only 0.4% of possible nucleotide mutations have a mutability that high. Inset shows the cumulative distribution functions for both spectra. Annotations in (A) show nucleotide substitutions in specific sequence contexts. (TIF) [file pcbi.1006981.s001.tif]

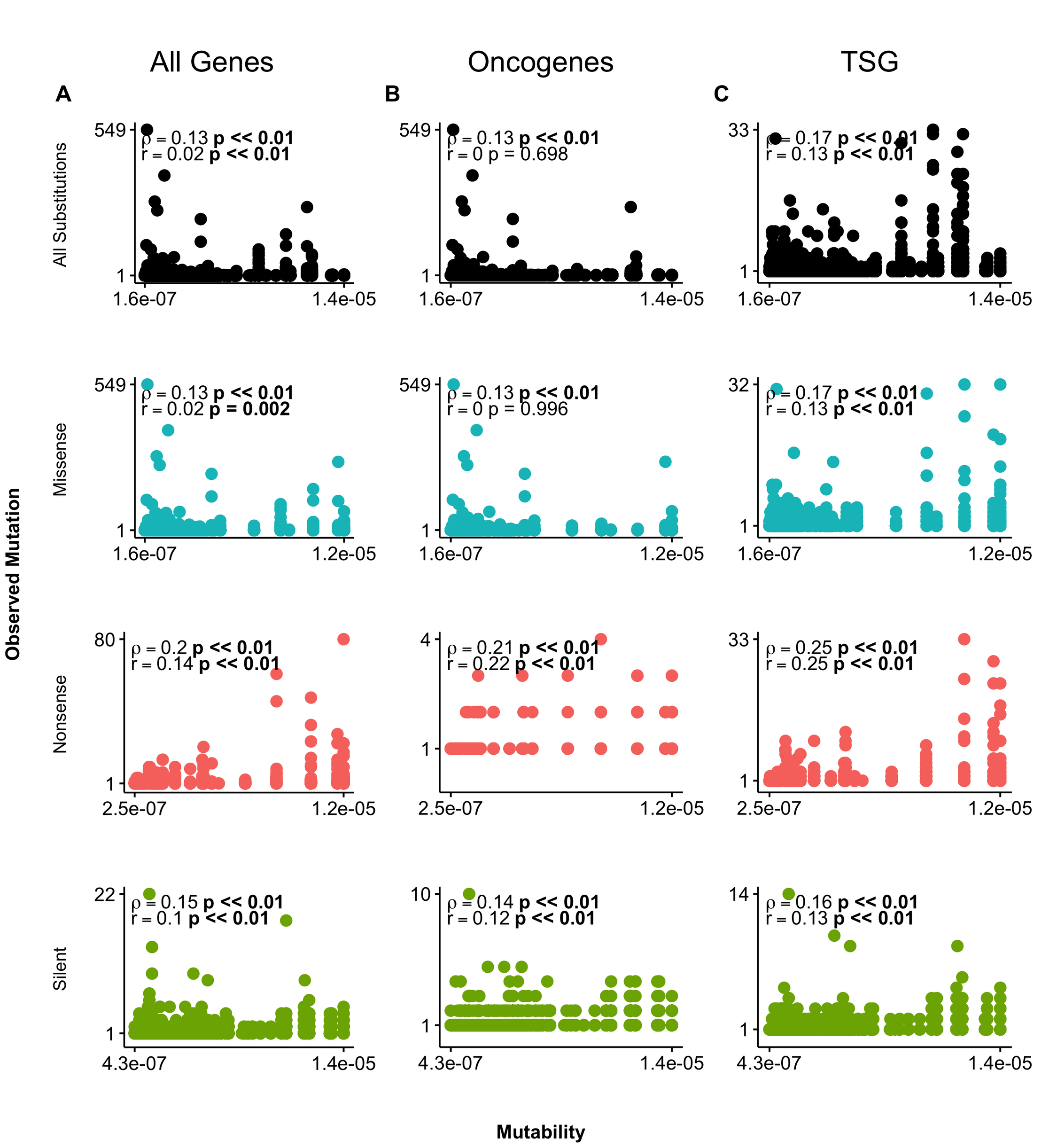

Supplement: S2 Fig — Scatterplots for (A) all cancer census genes (n = 520), (B) oncogenes (n = 202) and (C) tumor suppressor genes (TSG) (n = 166) for all mutation types: missense (blue), nonsense (red) and silent (green). Spearman and Pearson correlation coefficient with respective p-values are shown in all figures with p < 0.01 in bold. (TIF) [file pcbi.1006981.s002.tif]

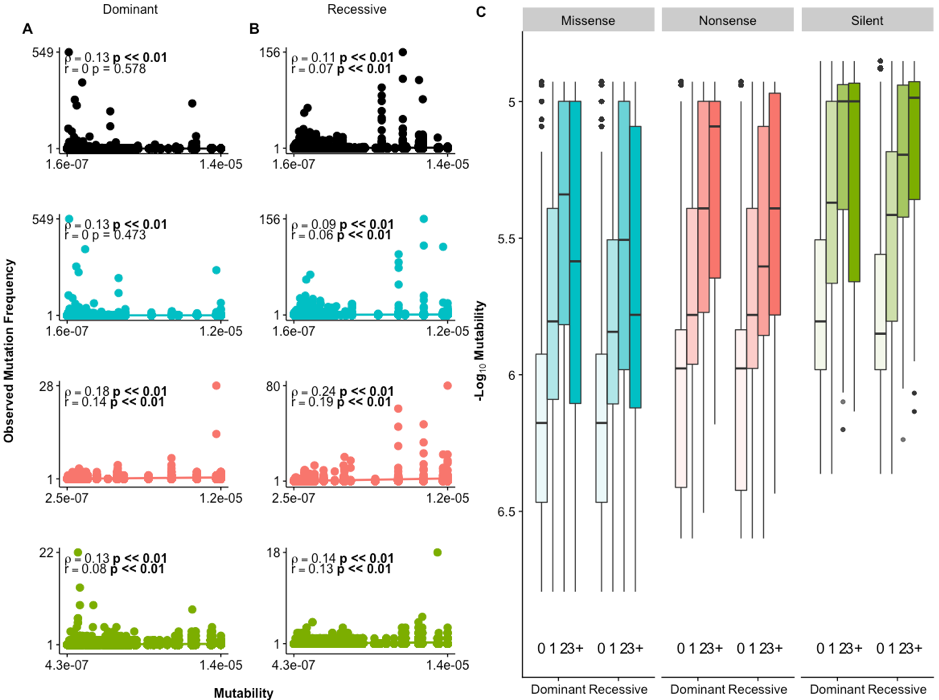

Supplement: S3 Fig — (A) Genes with only dominant mutations, (B) Genes with only recessive mutations. Different colors show scatterplots broken down by mutation type: missense (blue), nonsense (red) and silent (green). (C) Mutations in cancer census genes grouped by Dominant and Recessive mutations. Spearman and Pearson correlation coefficient with respective p-values shown in all, significant at p < 0.01 in bold. Counts summarized in Table S1. (TIF) [file pcbi.1006981.s003.tif]

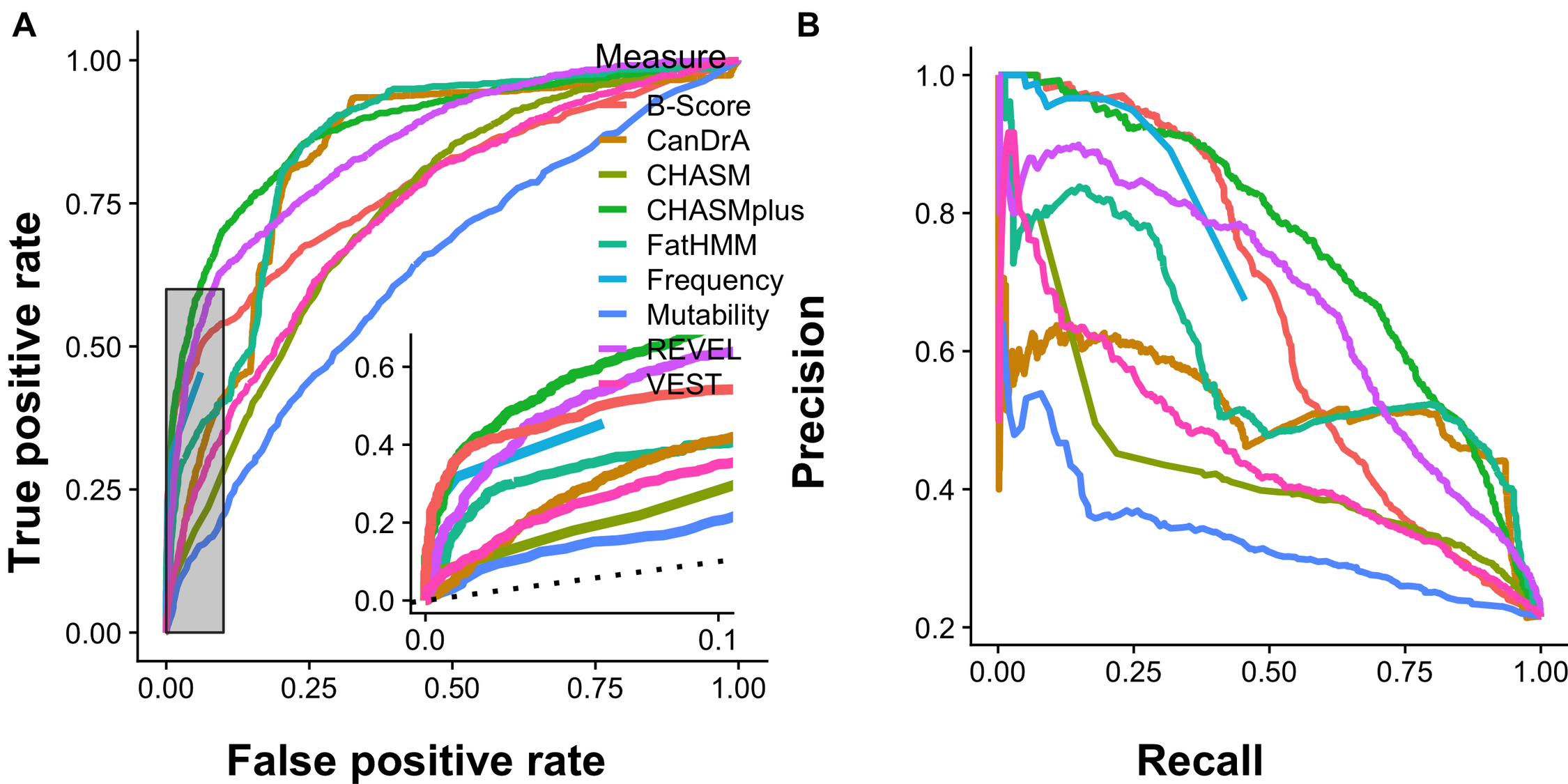

Supplement: S4 Fig — (A) ROC curves for B-Score, and observed mutational frequency based on mutation frequency in COSMIC v85 cohort. Inset shows the performance of highlighted area corresponding to up to 10% FPR. (B) Precision-recall curves for the same benchmark set. The ROC for reoccurrence frequency cannot be calculated for all mutations because some experimentally validated mutations were not observed in the COSMIC v85 cohort. (TIF) [file pcbi.1006981.s004.tif]

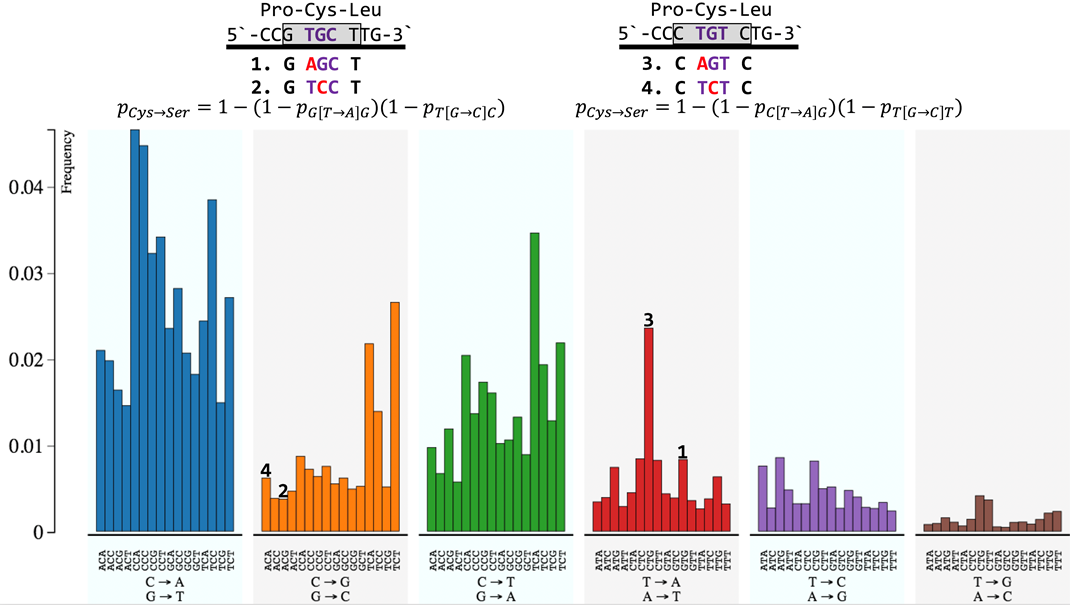

Supplement: S5 Fig — A peptide sequence of Pro-Cys-Leu could be encoded by nucleotide sequence CCG-TGC-TTG (left) or CCC-TGT-CTG (right). For both peptides, the pentanucleotides used to calculate the codon mutability for a Cys → Ser substitution has been highlighted in the blue box. Figure below shows lung adenocarcinoma cancer mutational profile used to calculate nucleotide mutability, x-axis is the 96 different possible context-dependent mutation types, y-axis shows mutation frequency. For each of the nucleotide mutations leading to a Cys → Ser amino acid substitution, the corresponding peak on the mutational profile is shown. (TIF) [file pcbi.1006981.s005.tif]

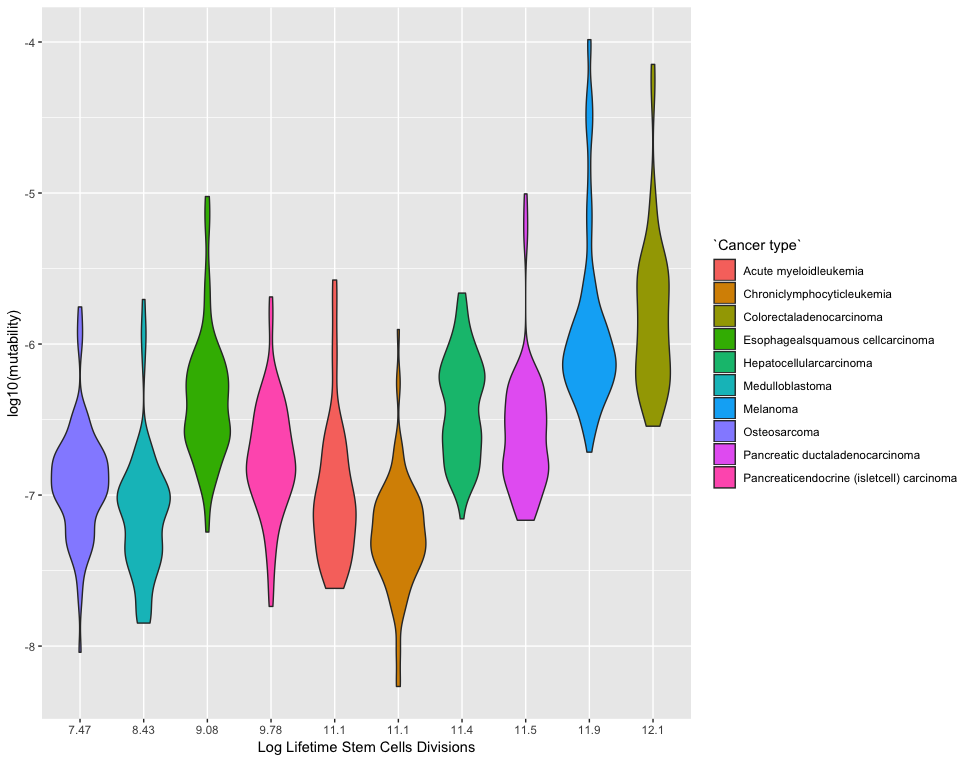

Supplement: S6 Fig — While there is a weak correlation between the two, it is not very prominent, and there is large variability in mutability values within the same cancer type. While Tomasetti and Vogelstein[56] established a strong correlation between the rate of stem cell divisions and cancer risk across various tissues, but it has been noted and discussed in multiple studies, that this correlation analysis did not include many cancer types and did not account for age-related variations in the numbers of stem cell divisions and large variations in cancer risks in different population groups. The estimates of mutation rates per generation per site (~10e-9) are averaged over all cell divisions and all genomic sites, while mutation rate can vary two orders of magnitude depending on the site location in genome and protein sequences. (TIF) [file pcbi.1006981.s006.tif]
